# Comparing robot-assisted versus laparoscopic Ladd's procedure in children with congenital intestinal malrotation

**DOI:** 10.1007/s13304-025-02177-2

**Published:** 2025-04-15

**Authors:** Ken Chen, Shuhao Zhang, Qingjiang Chen, Zhigang Gao

**Affiliations:** Department of General Surgery, Children’S Hospital, Zhejiang University School of Medicine, National Clinical Research Center for Child Health, Hangzhou, 310051 Zhejiang Province China

**Keywords:** Congenital intestinal malrotation, Children, Ladd's procedure, Laparoscopy, Robot-assisted

## Abstract

The aim of this study was to perform a comparative analysis of robot-assisted versus laparoscopic Ladd's procedure on peri- and postoperative outcomes. All Ladd’s procedures performed on patients with congenital intestinal malrotation between January 2020 and December 2023 were identified. Peri- and postoperative data were collected and compared between robot-assisted and laparoscopic groups. Fifty-seven robot-assisted and 38 laparoscopic Ladd’s procedure cases were identified and compared for outcomes. No robotic cases were converted to open procedure, while four laparoscopic cases were converted to open procedure (*p* = 0.048). Although robotic cases suffered higher hospitalization costs (*p* < 0.001), the postoperative complication rate was lower for the robotic group compared to the laparoscopic group (*p* = 0.038). Robot-assisted Ladd's surgery is safe and effective for the treatment of congenital intestinal malrotation in children, reducing the difficulty of surgery, but at increased cost.

## Introduction

Congenital intestinal malrotation refers to a malformation of the digestive tract that occurs during embryonic intestinal development due to the atypical rotation of the superior mesenteric artery at its axis. Pediatric duodenal obstruction is frequently caused by malrotation, with an incidence rate ranging from 0.2 to 1% [1, 2]. Due to the potential benefits of being minimally invasive with less pain and rapid recovery, an increasing number of surgeons prefer the laparoscopic Ladd’s procedure over the open procedure in treating congenital intestinal malrotation. With the development of minimally invasive surgical technology, the da Vinci robotic surgical system has been effectively utilized to treat certain malformations of digestive system in children, such as congenital megacolon, congenital choledochal cyst, and duodenal stenosis, with favorable outcomes [3, 4]. It has also been used to successfully treat adult cases with intestinal malrotation [5], but no studies regarding its application in children have been reported. This study aimed to compare peri- and postoperative outcomes after robot-assisted and laparoscopic Ladd's procedure.

## Methods

### Research design

All Ladd’s procedures performed on patients aged up to 18 years with congenital intestinal malrotation at the Children's Hospital of Zhejiang University School of Medicine between January 2020 and December 2023 were identified and a retrospective study was performed based on the clinical data. Patients with significant abnormalities of the heart and lung, omphalocele, gastroschisis, and intestinal atresia, with signs of intestinal strangulation and necrosis and with previous abdominal surgery history were excluded. This study was approved by the Medical Ethics Committee of the Children's Hospital of Zhejiang University School of Medicine (No. 2022-IRB-067). The parents of all enrolled children voluntarily chose which surgical plan was used and signed the informed consent for surgery.

All robotic surgeries were performed using the da Vinci Xi surgical system and all robot-assisted and laparoscopic Ladd's surgeries were completed by the same surgical team. Factors compared between patients who underwent robot-assisted versus laparoscopic Ladd’s procedure included demographic characteristics, clinical outcome and operative time, conversion to open surgery, length of stay, postoperative feeding time, complications, degree of volvulus, secondary surgery and hospitalization costs including examination fees, medication costs, and surgical fees.

### Robot-assisted and laparoscopic Ladd’s procedures

#### Robot-assisted Ladd's procedure

**Fig. 1:**
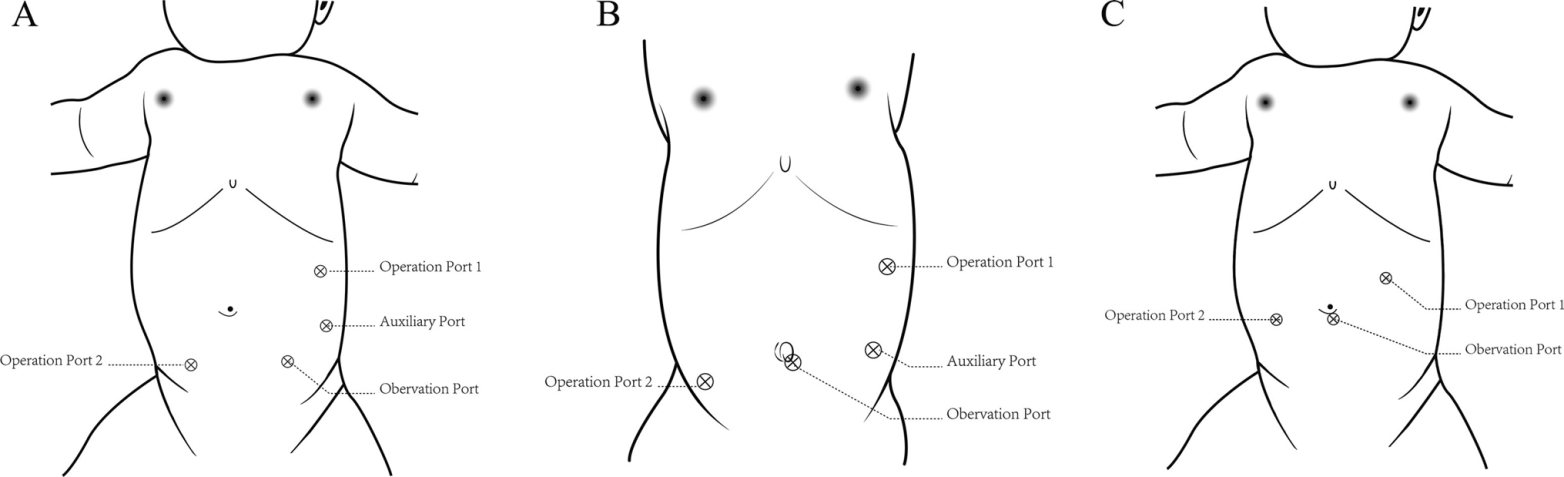
The port position of da Vinci robot-assisted and laparoscopic Ladd's surgery. **A** Newborn, **B** non-newborn, **C** laparoscopy

After general anesthesia, the patient was placed in a supine position with the head high and the feet low tilted approximately 30°. Port placements are shown in Fig. 1A, B. The assistant operated the laparoscopic instruments through the auxiliary port to help expose the field of vision and to transfer items.

**Fig. 2:**
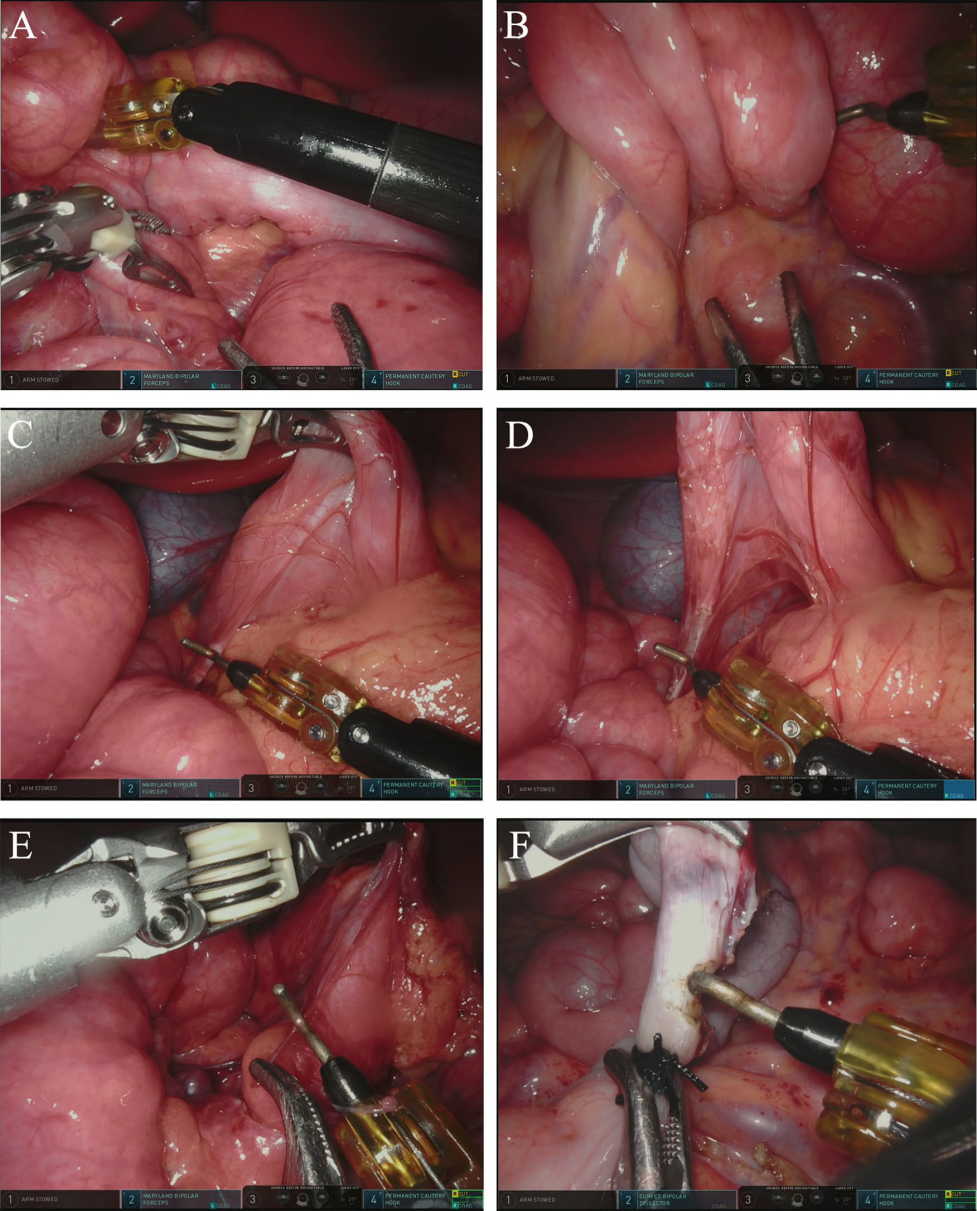
The surgical procedures of da Vinci robot-assisted Ladd's surgery: **A** finding the ileocecal junction, **B** reversing the twisted intestine, **C** releasing the Ladd's bands, **D** releasing the adhesions around the duodenum to the proximal jejunum, **E** widening the mesenteric root, **F** removing the appendix

Depending on the size of the abdominal cavity of the child, the hepatic round ligament could be suspended to increase the operating space. The position of the ileocecal junction is ascertained and the small intestine or mesentery is alternately pulled from the ileocecal junction to the proximal end until reaching the duodenum. Reduction of intestinal volvulus was performed and any adhesions encountered during the process were separated. Ladd's bands extending from the cecum or ascending colon to the right upper quadrant were loosened by electrocautery hook. All adhesions around the duodenum to the proximal jejunum are loosened to relieve duodenal compression and then straighten the duodenum. The abnormal adhesion at the root of the mesentery was released and the relatively narrow mesentery was fully expanded. After the appendix was removed, the duodenum and proximal jejunum were placed in the right abdomen and the cecum and ascending colon in the left abdomen. The stomach was inflated with a gastric tube to check whether the duodenum and proximal jejunum were unobstructed. After checking for remaining abnormalities, the endoscope was removed, the incision sutured and the operation completed, as seen in Fig. 2A–F.

#### Laparoscopic Ladd's operation

The surgical steps are the same as those for the da Vinci robot-assisted Ladd’s procedure. Port placements are shown in Fig. 1C.

### Statistical analysis

Continuous variables were described using median and interquartile range (IQR) and categorical variables were described using number and percentage. The Mann–Whitney U and Pearson chi-square tests were used to compare continuous variables and categorical variables for robot-assisted versus laparoscopic Ladd’s procedures. All analyses were performed using SPSS 22.0. *p* < 0.05 was considered statistically significant.

## Results

### Patient characteristics and perioperative data

Of the 95 children who met the inclusion criteria, 57 underwent a robot-assisted Ladd’s procedure and 38 underwent a laparoscopic Ladd’s procedure. In the robotic group, 53 cases of children presented with repeated vomiting and 4 cases with abdominal pain. In the laparoscopic group, 34 cases of children had repeated vomiting and four cases had abdominal pain.

All the children were diagnosed by ultrasound or upper gastrointestinal radiography. In the robotic group, 55 cases were diagnosed by ultrasound and two cases by upper gastrointestinal radiography. In the laparoscopic group, 35 cases were diagnosed by ultrasound and three cases by upper gastrointestinal radiography. All patients were intraoperatively diagnosed with congenital intestinal malrotation, which did not show significant differences 540 degrees (360–540) vs. 360 degrees (360–540), *p* = 0.1073.

Demographics and perioperative characteristics were similar between the two groups, as seen in Table 1. There was no conversion to open procedure in the robotic group, while four cases in the laparoscopic group were converted to open procedure. Two cases were due to dense adhesions at the mesenteric root with obvious intestinal distension, one case due to difficulty exposing the duodenum and incomplete release of Ladd's bands resulting in mesenteric edema and one case due to severe bleeding of the mesentery with difficulty in hemostasis.

**Table 1:** Demographics and perioperative characteristics of patients who underwent robot-assisted and laparoscopic Ladd’s procedure

	Robotic group (*n* = 57)	Laparoscopic group (*n* = 38)	*p* value
Age, months (IQR)	0.4 (0.3–1.0)	0.5 (0.3–25.5)	0.399
Gender (male: female)	43:14	28:10	0.847
Weight, Kg (IQR)	3.2 (2.9–4.0)	3.3 (2.9–13.2)	0.408
Surgical time, min (IQR)	86(74.5, 105.5)	89 (65.8, 116.3)	0.843
Conversion to open procedure, *n* (%)	0 (0.0%)	4 (10.5%)	0.048
Degrees of volvulus	540 (360–540)	360 (360–540)	0.1073
Symptom			0.821
Abdominal pain (*n*)	4	4	
Vomiting (*n*)	53	34	
Methods of diagnosis			0.639
Ultrasound (*n*)	55	35	
Upper gastrointestinal radiography (*n*)	2	3	

### Robot-assisted and laparoscopic Ladd’s procedure: outcomes (Table 2)

Patients undergoing robot-assisted Ladd’s procedure had higher mean hospitalization costs of 9132.39 USD compared with 3517.95 USD (*p* < 0.001). Postoperative feeding time and postoperative LOS of patients undergoing two procedures were comparable. The median follow-up time was 6 months with an IQR of 4.5 to 8 after discharge. Postoperative complications occurred were 3.5% in two robotic cases and 18.4% in seven laparoscopic cases (*p* = 0.038).

**Table 2:** Outcomes of patients who underwent robot-assisted and laparoscopic Ladd’s procedure

	Robotic group (*n* = 57)	Laparoscopic group (*n* = 38)	*p* value
Postoperative feeding time, days (IQR)	4 (3, 6)	4 (3, 6)	0.261
Postoperative LOS, days (IQR)	10 (7, 13)	10.5 (7.8, 15)	0.174
Hospitalization costs, USD (IQR)	9132.39 (8625.48, 10,125.84)	3517.95 (2731.36, 4091.62)	< 0.001
Secondary surgery, *n* (%)	1 (1.8%)	3 (7.9%)	0.348
Any postoperative complications, *n* (%)	2 (3.5%)	7 (18.4%)	0.038
Recurrent volvulus	0 (0.0%)	2 (5.3%)	0.157
Adhesive ileus	1 (1.8%)	2 (5.3%)	0.719
Chylous ascites	1 (1.8%)	3 (7.9%)	0.348

During hospitalization and follow-up, the robotic group had two cases of postoperative complications (chylous ascites and adhesive bowel obstruction), with one case requiring a second surgery due to adhesive bowel obstruction. The laparoscopic group had seven cases of postoperative complications, with three cases requiring a second surgery. There were three cases of chylous ascites, two cases of adhesive bowel obstruction of which one required a second surgery and two cases of recurrent volvulus both requiring second surgeries. Neither group of patients suffered complications such as wound infection, wound dehiscence, or incisional hernia postoperatively. The specific details and treatment of patients with postoperative complications are displayed in Table 3.

**Table 3:** Specific details and management of patients with postoperative complications in robotic group and laparoscopic group after operation

No	Group category	Gender	Age (M)	Postoperative complication	Treatment and result
1	robot	Male	0.5	Adhesive ileus	The secondary surgery was performed 2 weeks after the initial surgery. During the surgery, it was found that the distal duodenum was partially adherent, resulting in obstruction
2	robot	Male	1.2	Chyle ascites	Patient was discharged with a week of low-fat milk powder intake at home. Follow-up abdominal ultrasound confirmed complete resolution of ascites, and the abdominal drainage tube was subsequently removed in the outpatient clinic
3	laparoscopy	Male	0.7	Adhesive ileus	Following a 1-week course of fasting, gastrointestinal decompression and parenteral nutrition, the child recovered
4	laparoscopy	Male	1.1	Adhesive ileus	This patient had the second surgery five days after being discharged, which was 12 days after the initial surgery. During the surgery, it was found that the Ladd's bands near the duodenum were not completely loosened, causing pressure on the intestinal tract
5	laparoscopy	Male	1.5	Chyle ascites	Patient was discharged with 2 weeks of low-fat milk powder intake at home. Follow-up abdominal ultrasound confirmed complete resolution of ascites, and the abdominal drainage tube was subsequently removed in the outpatient clinic
6	laparoscopy	Female	65	Chyle ascites	Following a 1-week course of fasting and parenteral nutrition, followed by an additional week of low-fat diet, abdominal ultrasound revealed a reduction in ascites volume. At 1-month outpatient follow-up, abdominal ultrasound demonstrated complete resolution of ascites
7	laparoscopy	Male	129	Recurrent volvulus	This patient had the second surgery 2 days after being discharged, which was 8 days after the initial surgery. During the surgery, it was found that the small intestine was clockwise twisted by 360°, and the mesenteric root was adherent. The child recovered well after the surgery, and the abdominal ultrasound examination was normal 2 weeks later
8	laparoscopy	Female	102	Recurrent volvulus	A secondary surgery was performed 1 week after the initial surgery. During the surgery, it was found that the small intestine was clockwise twisted by 540°, the proximal jejunum was adherent to the ileocecal junction, and the narrow mesentery root was not fully expanded
9	laparoscopy	Male	37	Chyle ascites	Following a 1-week course of fasting and intravenous hyperalimentation, followed by an additional week of low-fat diet, abdominal ultrasound demonstrated complete resolution of ascites

## Discussion

Since the first report of laparoscopic Ladd's surgery for congenital intestinal malrotation in 1995, it has been adopted due to its advantages of small incisions, rapid recovery of intestinal function after surgery, and short hospitalization time compared to open surgery [6–8]. However, due to the limitations of the operational stability, flexibility, and two-dimensional surgical view of laparoscopic equipment, the operation of reversing twisted intestinal tubes under laparoscopy is quite difficult and especially in newborns where the narrow operating space is a major challenge even for skilled laparoscopic surgeons [9]. Hsiao's research showed that the rate of laparoscopic conversion in newborns was as high as 50%, while it was only 18% in older children [10].

In this study, the conversion rate in the laparoscopic group was 10.5%, which was comparable to previous studies in the past three years [8, 11]. A meta-analysis of screened papers published from 1995 to 2019 by Karina et al. showed that there were 11 cases of laparoscopic Ladd's surgery converted to open surgery among 95 cases, with a conversion rate of 11.1%. Among them, the most common reason was the insufficient surgical view of key structures, accounting for 54.5%, followed by lack of surgical experience and technical problems of the operator [12]. In this study, there were four cases of laparoscopic conversion to open surgery, of which two were related to intestinal distension significantly affecting the surgical field and causing difficulty resetting intestinal torsion due to dense adhesion of the mesenteric root. One case was related to difficulty in exposing the duodenum due to mesenteric edema affecting the surgical field, resulting in incomplete release of Ladd's bands and one was related to excessive bleeding during the expansion of mesenteric root due to vascular injury, indicating that surgical view is the main reason for conversion in laparoscopic Ladd's surgery. However, there were no cases of conversion in the robot group, which the author believes is mainly due to the characteristics of da Vinci robotics.

Compared with laparoscopic surgery, robotic surgery has advantages such as a robotic arm with seven degrees of freedom for rotation, high-resolution 3D surgical view and tremor filtering [13, 14]. The Ladd's bands in children with intestinal malrotation begin at the cecum and proximal colon, wrapping and compressing the duodenum until it ends at the right upper quadrant peritoneum. The position is deep and traditional laparoscopic exposure and release are difficult. The robotic surgery system can provide high-definition 3D images with a magnification of 10 to 15 times, which is far superior to laparoscopic surgery with a magnification of three to four times. It can more clearly and stereoscopically display Ladd's bands and surrounding adjacent organs and tissues, allowing for clearer identification of blood vessels during expansion of the mesenteric root to avoid injury and also allowing for timely detection of bleeding sites to achieve hemostasis. The robotic arm of the da Vinci robot can rotate freely in a clockwise or counterclockwise direction at 360 degrees and can filter tremors, allowing for more flexible and precise operations in confined spaces, which is helpful for completing intestinal torsion resetting and can also smoothly release severe or deep adhesions. The second reason is targeted port placement and intraoperative suspension, which increases the operating space in the abdominal cavity. Finkelstein et al. believed that the limited space inside a small baby can severely hinder the movement of the robotic arm, resulting in a high probability of instrument collision, which can affect intraoperative operations and even lead to conversion [15]. For newborns, the experience of this study is that the robotic surgery port placement is targeted at the targeted upper right abdomen using a semi-arc port placement, while intraoperative suspension of the hepatic round ligament through the abdominal wall is used to increase the operating space of the robotic arm. Several studies have also shown that for complex abdominal surgery, the learning curve of da Vinci robotic surgery is significantly shorter than laparoscopic surgery, making it easier for the surgeon to adapt and master [16, 17].

Compared with open surgery, laparoscopic Ladd's procedure does not require exposure of the intestinal tract in air [18], which has less interference with the intestinal tract. The incision of laparoscopic surgery is smaller and children have less postoperative pain and can practice walking exercises early after surgery, which is beneficial for promoting the recovery of intestinal function and significantly shortening the time to first feeding and hospitalization after surgery [8, 19]. In this study, there was no particular advantage of the robot group in terms of postoperative feeding time and hospitalization time compared with the laparoscopic group. The processing methods and steps for the intestinal tract in both surgical procedures were completely consistent, and both operations were completed under laparoscopy to achieve resetting of intestinal twisting, release of Ladd's bands and the adhesions around the jejunum, and resection of the appendix.

There was no significant difference in operation time between the two groups. Although the robotic surgery system is more accurate in separating, dissecting, and releasing structures adjacent to the duodenum and mesenteric root, with less intraoperative bleeding, which reduces the operation time in the abdominal cavity, the average installation time of the da Vinci robotic system is about 15 min, so there was no significant difference in total operation time between the two groups.

In terms of postoperative complications, this study showed that both groups had complications of adhesive ileus, recurrent intestinal twisting, and chylous ascites. There were no incision infections or tears in either group. The overall incidence of postoperative complications in the robot group was significantly lower than that in the laparoscopic group. This may be related to the inherent advantages of the robotic surgery system, which helps operators to expose the duodenum smoothly, clearly identify and completely release the Ladd's bands, accurately widen the mesenteric root, and effectively avoid injury to the intestinal tract, mesenteric vessels, and lymphatic vessels, reducing the risk of adhesive ileus and chylous ascites [20]. Some scholars believe that intra-abdominal intestinal adhesions can reduce the risk of recurrent intestinal twisting after surgery, but this view is still controversial [21, 22].

This study showed that there were two cases of recurrent intestinal twisting in the laparoscopic group and both cases were found to have narrowed mesenteric roots during secondary surgery. Compared with laparoscopic surgery, the incidence of intestinal adhesions in the robot group was lower, but there was no case of intestinal twisting. This suggests that adhesion is not a key factor in preventing recurrent intestinal twisting and it is hypothesized that the key is fully widening the narrowed mesenteric root during the operation. However, this study is a retrospective non-randomized controlled study with a small sample size and short follow-up time, so this point needs to be further confirmed by future studies.

In addition to the two cases of recurrent intestinal volvulus requiring secondary surgery in the laparoscopic group, there was also one case of intestinal obstruction due to incomplete Ladd's bands release requiring secondary surgery. The cause may be related to incomplete separation of the tightly adherent Ladd's bands by the surgeon during the operation, while trying to avoid excessive separation and tissue injury. There was also one case of obstruction due to local adhesion of the distal duodenum in the robot group requiring secondary surgery. The cause may be related to the lack of tactile feedback in the robotic surgical system, which led to the injury of the intestinal seromuscular layer during the process of grasping and pulling the intestine by the surgeon [13, 14].

Although robotic surgery systems have many advantages compared to traditional laparoscopic surgery, they also have some disadvantages. The trocar used by the robot is not adapted for children and the 8 mm trocar aperture is too large. Due to the need for auxiliary operation ports, the number of trocar in robotic surgery is more, so the aesthetic quality of the abdominal wall incision is not as good as laparoscopic surgery. Robotic surgery is expensive and this study showed that in cases with similar postoperative hospitalization time, the hospitalization cost of the robot group was significantly higher than that of the laparoscopic group.

The present study has some limitations including its retrospective nature, single center, selection bias due to non-randomization, and short follow-up time, so future studies need to involve larger randomized samples, multiple centers, and long follow-up periods.

## Conclusion

Compared to laparoscopic Ladd's surgery, the da Vinci robot-assisted Ladd's surgery shows a trend for a lower rate of conversion and complications, but the cost is higher. As a micro-innovation technology, it is safe and effective for treating congenital intestinal malrotation in children, with good prognosis and is worth further application.

## Data Availability

The datasets used and/or analysed during the current study are available from the corresponding author on reasonable request.
